# Properties of Emulsion Co-Precipitated Collagen/Bambara Groundnut Protein-Based Film as Influenced by Basil Essential Oil and Soy Lecithin

**DOI:** 10.3390/polym17091139

**Published:** 2025-04-22

**Authors:** Md. Shihabul Awal, Soottawat Benjakul, Thummanoon Prodpran, Krisana Nilsuwan

**Affiliations:** 1 International Center of Excellence in Seafood Science and Innovation (ICE-SSI), Faculty of Agro-Industry, Prince of Songkla University, Hat Yai 90110, Songkhla, Thailand; shihab@hstu.ac.bd (M.S.A.); soottawat.b@psu.ac.th (S.B.); thummanoon.p@psu.ac.th (T.P.); 2 Department of Food and Nutrition, Kyung Hee University, Seoul 02447, Republic of Korea; 3 Center of Excellence in Bio-Based Materials and Packaging Innovation, Faculty of Agro-Industry, Prince of Songkla University, Hat Yai 90110, Songkhla, Thailand

**Keywords:** film-forming emulsion, oil droplet, basil essential oil, soy lecithin, emulsified film

## Abstract

Protein-based films have gained attention due to their potential as biodegradable packaging. This study investigated the properties and characteristics of film-forming emulsions (FFEs) and their films based on co-precipitated protein (CPP) from Bambara groundnut protein isolate (BGPI) and acid-soluble collagen (ASC) emulsified with different levels of basil essential oil (BE) (50%, 75% and 100%) and soy lecithin (SL) (25% and 50%). The oil droplet size, stability, and distribution of FFEs were characterized. Larger oil droplet sizes, a higher flocculation factor, and a higher coalescence index were observed for FFEs emulsified with higher levels of BE and SL. All FFEs had uniform oil distribution. Films from different FFEs were formed and analyzed. Films containing BE and SL had higher thickness, elongation at break, b*-value, water vapor and UV-light barrier properties, but a lower tensile strength than the control film. Emulsion films exhibited smooth surface and rough cross-section and were heat-sealable. FTIR spectra indicated lower protein interactions in the emulsion film containing higher levels of BE and SL. The film containing 100% BE had the highest antioxidant activities, regardless of the SL level used. The emulsification of BE and SL at various levels thus influenced the properties and characteristics of the FFE and emulsion film.

## 1. Introduction

Non-biodegradable plastics derived from fossil fuels have become increasingly prevalent as packaging materials due to their cost-effectiveness compared to renewable polymers [1]. However, the environmental concerns associated with fossil fuel-based plastics and the limited availability of petroleum resources have challenged the researchers to explore biodegradable polymers made from renewable resources as a sustainable alternative [2]. Protein extracted from different sources, such as fish skin acid-soluble collagen (ASC) [3], tilapia skin gelatin [4], Bambara groundnut protein isolate (BGPI), and wheat protein [5,6], are notable for their good film-forming properties, biocompatibility, and biodegradability over non-degradable polymers. The use of BGPI and ASC alone still has shortcomings, such as low mechanical and water barrier properties [7]. Therefore, the co-precipitation or blending of different proteins to improve barrier and mechanical properties was proposed [7].

Nowadays, active packaging films, in which natural polymers are mixed with functional compounds, have established themselves as safe and beneficial alternative for non-biodegradable packaging [8]. The European Union (EU) Commission defines active packaging as any substance or device that extends shelf life or enhances the packaging environment [9]. An improvement of the water vapor barrier properties could be achieved by incorporating hydrophobic substances such as essential oils, edible oils, fatty acids, and wax into protein-based films [10]. Protein-lipid blended films with good water vapor barrier properties are produced by incorporating lipids through emulsification. Additionally, smaller lipid particle sizes also contribute to the reduction in the water vapor barrier properties [8].

Essential oils, derived from various plant parts, are complicated composites of secondary metabolites that are volatile, including unsaturated and saturated hydrocarbons, alcohols, terpenes, aldehydes, and ketones [11]. The chemical composition of basil oils depends on the geography, variety, and extraction methods. Its major constituents included linalool, γ-bergamotene, eucalyptol, estragole, eugenol, methyl cinnamate, bicyclosesquiphellandrene, γ-cadinene, and germacrene D [12]. Linalool–eugenol oils with a high eugenol content showed good antioxidant activity. Oils rich in methyl chavicol and linalool exhibited the weakest activity. This was attributed to methylation blocking the OH group in compounds like methyl eugenol, thus reducing antioxidant activity [13].

The emulsified film’s attributes are related to the distribution and size of the oil droplets in the film [14]. The emulsion droplet size is reduced by mechanical equipment like homogenizers, microfluidizer, high-pressure homogenizers, ultrasound, etc. Ultrasound can induce physical, chemical, and mechanical changes in emulsion films, mainly because of the cavitation process that can break down the oil droplets. It has various applications in the chemistry, alimentary, pharmaceutical, and cosmetic fields [15]. Ultrasound improves the film microstructure by producing more regular, crystalline, and homogeneous surfaces and enhancing barrier and mechanical properties. Increasing the ultrasonication time and amplitude in emulsified hazelnut meal protein–clove essential oil-incorporated films reduced the zeta potential and particle size, as well as improved the WVP [16]. In addition, the use of different surfactants showed major consequences on the emulsions’ stability and the homogenous distribution of the oil droplets in the film matrix [17].

However, there are no reports on the emulsified CPP (co-precipitated protein) FFE and films with the incorporation of BE and SL at various levels in CPP films based on BGPI and ASC. Thus, the focus of the current study was to investigate the effects of basil essential oil and soy lecithin on the properties and characteristics of the CPP-based film-forming solution and emulsion films.

## 2. Materials and Methods

### 2.1. Chemicals and Reagents

Basil essential oil (*Ocimum basilicum*) with a relative density of 0.90 g/cm^3^ and purity of 100% was purchased from Botanicessence (Bangkok, Thailand). NaOH, acetic acid, HCl, methanol, and ethanol were obtained from Merck (Darmstadt, Germany). Trolox (6-hydroxy-2,5,7,8-tetramethylchroman-2-carboxylic acid) was purchased from Aldrich Chemical Company (Steinheim, Germany). Glycerol, Nile blue A, soy lecithin, sodium dodecyl sulfate (SDS), and TPTZ were bought from Fluka Chemicals (Buchs, Switzerland). DPPH, ferric chloride (FeCl_3_·6H_2_O), ferrous chloride (FeCl_2_), ABTS, potassium persulfate, ferrozine, and 2,2ʹ-azobis (2-amidinopropane) dihydrochloride (AAPH) were procured from Sigma-Aldrich, Inc. (St. Louis, MI, USA). Every chemical used in this investigation was of analytical grade.

### 2.2. Preparation of Bambara Groundnut Protein Isolate (BGPI)

The seeds of the Bambara groundnut (*Vigna subterranean*) were bought from Phatthalung, Thailand. After being dehulled, the dried seeds were smashed into a fine powder by a high-speed blender and sieved through a No. 60 mesh screen. The preparation of defatted Bambara groundnut powder (D-BGP) was carried out according to the instructions of Awal et al. (2024) [7]. To effectively disperse the powder, distilled water (DW) was added to D-BGP at a ratio of 1:100 (*w*/*v*) and shaken for 60 min. The pH was then adjusted to 11.0 and agitated for 60 min. The mixture was then centrifuged at 3000× *g* for 10 min at 25 °C (CR22N Hitachi, Tokyo, Japan). The collected supernatant pH was shifted to 4.5, shaken and centrifuged (3000× *g*, 10 min). After the addition of 10 volumes of DW to the precipitate, pH was shifted to 7.0. After centrifugation (3000× *g* for 10 min), the pellet was freeze-dried for 72 h at −50 °C using a ScanVac, Lynge, Denmark, freeze dryer. Until they were used, the resulting powders were kept at −40 °C. The resulting powder was called Bambara groundnut protein isolate (BGPI).

### 2.3. Extraction of Acid-Soluble Collagen (ASC) from Fish Skin

The skin of the sockeye salmon (*Oncorhynchus nerka*) was pretreated and defatted following the procedure outlined by Nilsuwan et al. (2022) [18]. About 50 g of pretreated skin was mixed with acetic acid (0.5 M) at a ratio of 1:50 (*w*/*v*) and the mixture was agitated continuously for 48 h at 4 °C. The mixture was then passed through cheesecloth for filtration. Collagen was precipitated by adding NaCl to obtain a final concentration of 2.6 M NaCl and the pH was brought to 7.5. Collagen was collected using a Hitachi CR22N centrifuge (Tokyo, Japan) (10,000× *g* for 5 min) at a refrigerated temperature. After washing the collagen pellet with DW at a ratio of 1:50 (*w*/*v*), it was stirred for 30 min and centrifuged (10,000× *g*, 4 °C, 5 min). The desalting and centrifugation procedures were carried out twice and the collected pellet was freeze-dried, as previously stated. The dried powder was called acid-soluble collagen (ASC).

### 2.4. Co-Precipitated Protein (CPP) Preparation

The CPP was prepared by the co-precipitation of BGPI and ASC at a ratio of 25:75, as outlined by Awal et al. (2024) [7]. The mixture of BGPI and ASC (1%, *w*/*v*) was individually prepared, mixed with DW, and the pH was shifted to 3.0. The solutions were swirled vigorously for 24 h at 4 °C, then mixed at a 25:75 (*v*/*v*) ratio of BGPI/ASC and swirled for another 60 min at 4 °C. The mixed solution’s pH was adjusted to 7.0 and shaken for 60 min. The pellets were prepared using a Hitachi CR22N centrifuge (Tokyo, Japan) (10,000× *g*, 60 min) at a refrigerated temperature. As mentioned above, a freeze dryer was used to freeze-dry the co-precipitated (CPP) pellet and samples were analyzed.

### 2.5. Preparation of the Film-Forming Emulsion

To obtain a protein content of 2% (*w*/*v*), CPP was mixed with acetic acid (1%, *v*/*v*) and shaken constantly at 4 °C for 60 min. To prepare a film-forming solution (FFS), 25% glycerol (*w/w*, according to CPP content) was included as a plasticizer and the mixture was shaken for 30 min. In order to produce film-forming emulsion (FFE), soy lecithin (SL) and basil essential oil (BE) were mixed at 25% and 50% (*w/w*, based on BE content) before being added to FFS at 50%, 75%, and 100% (*w/w*, based on the CPP content) [19]. An IKA Labortechnik Homogenizer (Selangor, Malaysia) was then used to homogenize the mixture at 22,000 rpm for 3 min. To prepare a fine emulsion, the coarse emulsion was sonicated for 10 min with an ultrasonic processor (Model VC750, Newtown, CT, USA) at 70% amplitude and a 5 s pulse duration. The FFS without BE and SL was prepared in the same manner to obtain the control film. All FFE samples were analyzed for oil stability and the droplet size, and they were also used for film formation.

### 2.6. Analysis of the Film-Forming Emulsion

#### 2.6.1. Oil Droplet Size

The size of the oil droplets in the FFE was measured utilizing a zeta potential analyzer (ZetaPALs, Holtsville, NY, USA). Before analysis, the FFEs were mixed with a solution of SDS (1%, *w*/*v*) to disperse the droplets and avoid flocculation [19]. The following equations were used to calculated the surface-weighted mean (*d*_32_) and volume-weighted mean (*d*_43_) particle diameters, respectively:

(1) $$d_{32}=\frac{\sum n_{i}\;d_{i}^{3}}{\sum n_{i}\;d_{i}^{2}}$$

(2) $$d_{43}=\frac{\sum n_{i}\;d_{i}^{4}}{\sum n_{i}\;d_{i}^{3}}$$

where *n*_i_ represents the count of droplets within a specific size range and *d*_i_ corresponds to the diameter of these droplets.

#### 2.6.2. Flocculation Factor (F_f_) and Coalescence Index (C_i_)

To evaluate the flocculation factor (*F*_f_) and coalescence index (*C*_i_), distilled water was used to dilute FFEs both with and without a 1% (*w*/*v*) SDS solution before the measurement of the oil droplet size [19]. The calculations for *F*_f_ and *C*_i_ were performed employing the following equations:

(3) $$F_{f}=\frac{d_{43-\mathrm{SDS}}}{d_{43+\mathrm{SDS}}}$$

(4) $$C_{i}(\%)=\frac{\left(d_{43+\mathrm{SDS},\;t}-d_{43+\mathrm{SDS},\;\mathrm{in}}\right)}{d_{43+\mathrm{SDS},\;\mathrm{in}}}\;\times \;100$$

where the emulsion droplets’ volume-weighted mean particle diameters with and without the 1% SDS are indicated by the symbols *d*_43+SDS_ and *d*_43−SDS_, respectively. The term *d*_43+SDS, in_ represents the initial value of the volume-weighted mean particle diameter of the droplets when 1% SDS is present. Meanwhile, *d*_43+SDS, t_ represents the volume-weighted mean particle diameter of emulsion droplets containing 1% SDS after 24 h of storage.

#### 2.6.3. Confocal Laser Scanning Microscopy (CLSM)

The dispersion of oil droplets in film-forming emulsions was measured using a FV300 confocal laser microscope (Olympus, Tokyo, Japan) [19]. Before analysis, the FFE samples were dyed with Nile blue A at a ratio of 20:1 (*v*/*v*). A 50 µL of mixture was then put onto a microscopic slide and covered with a slip. For the lipid analysis, the fluorescence mode was employed and a helium–neon red laser with excitation and emission wavelengths of 543 nm and 561 nm, respectively, was used. The amplification was set at 200×.

### 2.7. Preparation of the Emulsion-Based Film

Before casting, the FFE was degassed under vacuum conditions (Wertheim, Germany) at room temperature for 30 min. Eight grams of FFE were cast into a square silicone resin plate (5 × 5 cm^2^) and air-blown for 12 h at room temperature and a relative humidity (RH) of 60 ± 5%. The film samples were then dried for 48 h at 25 °C and 50 ± 5% RH in a conditioned chamber. After being peeled off, the films were examined.

### 2.8. Analysis of the Films

#### 2.8.1. Thickness

A digital micrometer (Mitutoyo, Kawasaki-shi, Japan) was used for the measurement of the thicknesses of ten films at nine random spots. The mean thickness of the films was calculated.

#### 2.8.2. Mechanical Properties

The mechanical properties of the films, comprising tensile strength (TS) and elongation at break (EAB), were assessed according to the procedure described by Awal et al. (2024) [7]. Ten films (50 mm × 20 mm) were analyzed by a universal testing machine (Hampshire, UK) with a 200 N tensile load and speed of 30 mm/min. The films were clamped with an initial grip length of 40 mm.

#### 2.8.3. Water Vapor Permeability (WVP)

The WVP of the films was determined as described by Awal et al. (2024) [7]. Film samples (35 mm× 35 mm) were put on the top of aluminum permeation cups comprising dried silica and sealed securely. Then, the sealed cups were retained in a conditioned chamber at room temperature and 50% relative humidity, and the cups were weighed every hour for a maximum of 10 h. The WVP was then computed.

(5) $$\mathrm{WVP}\;(\frac{gm}{m^{2}s\;Pa})=\frac{w\;l}{A\;t\;(P_{2}-P_{1})}$$

where *w* is the weight increase of the aluminum cup (g), the film thickness is denoted as *l* (m), *A* denotes the exposed film area (m^2^), t is the weight gain time (s) and *P*_2_ − *P*_1_ is the variation in vapor pressure (1583.7 Pa) throughout the film at 25 °C.

#### 2.8.4. Color and Light Transmission

The L*, a*, b*, and ΔE* values of films were estimated using a CIE colorimeter (Hunter Associ. Lab., Reston, VA, USA) as per Awal et al. (2024) [7]. The device was standardized with black and white plates before testing. ΔE* was computed using the equation as follows:

(6) $$\Delta E*=\sqrt{{\left(\Delta L*\right)}^{2}+{\left(\Delta a*\right)}^{2}+{\left(\Delta b*\right)}^{2}}$$

where ΔL*, Δa*, and Δb* represent the differences between the film sample’s color parameters and those of the standard white plate (L* = 92.81, a* = −1.24, and b* = 0.53).

According to the method of Awal et al. (2024) [7], a Shimadzu UV-vis spectrophotometer (UV-1800, Kyoto, Japan) was utilized to evaluate the light transmission of films in the UV-vis ranges of 200–800 nm.

#### 2.8.5. Scanning Electron Microscopy (SEM)

The microstructure of the selected films was examined [20] employing a scanning electron microscope (Quanta 400, Eindhoven, The Netherlands). Under liquid nitrogen, the film samples were fractured to observe the film cross-section. Subsequently, the specimens were placed on bronze stubs and coated with a gold layer using a sputter coater to enhance sample conductivity. A 20 kV acceleration voltage and 5000× magnification for surface images and 1800× for cross-section images were used to take the pictures.

#### 2.8.6. Sealing Ability

Seal strength and efficiency were evaluated as outlined by Tongnuanchan et al. (2016) [20]. Strips of experimental films were cut to 25 mm × 20 mm. Two cut film strips were lined up face-to-face together and sealed using a ME 300HIM sealer equipped with a magnet (Nonthaburi, Thailand). The sealing process involved heating them at a temperature of 150 ± 0.5 °C for 6 s of heating and 6 s of cooling. The width of sealed region was two millimeters. All sealed films were conditioned for 48 h at 25 ± 0.5 °C and 50 ± 5% relative humidity before evaluation. The peel test was used to evaluate the seal strength and efficiency according to the ASTM F-88 [21] standard by a universal testing machine with a few adjustments (Hampshire, UK) at room temperature and 50 ± 5% RH. The film previously sealed was attached at both ends to a 200 N static load cell of the device, which was positioned perpendicular to the direction of tension. The gap between the clamps was 50 mm. The tensile load was applied to the films until seal failure. Seal strength and seal efficiency were computed using following equations:

(7) $$\mathrm{Seal}\;\mathrm{strength}\;(N/m)=\frac{Peak\;force}{Film\;width}$$

(8) $$\mathrm{Seal}\;\mathrm{efficiency}\;(\%)=\frac{Peak\;force}{Tensile\;force}\times 100$$

where peak force (N) is the highest force recorded during a seal test and tensile force is the force (N) recorded during a tensile strength test.

#### 2.8.7. FTIR

Prior to the study, the films were stored in a desiccator for two weeks at ambient temperature to dehydrate and condition the films. The films were analyzed using an FTIR spectrometer from Bruker (Ettlinger, Germany) outfitted with an ATR diamond cell from PIKE Technology Inc. (Madison, WI, USA) [7]. With a resolution rate of 4 cm^−1^ in the wavenumber range of 400–4000 cm^−1^, 32 scans were carried out. The background signal was subtracted from all samples by the program OPUS 3.0 (Bruker, Ettlingen, Germany).

### 2.9. Antioxidant Activities of Films

Initially, film samples (100 mg) were cut into smaller pieces and subjected to continuous stirring in 10 mL of 95% methanol in an amber bottle that had been tightly capped overnight. Subsequently, the resulting mixtures were centrifuged at 2800× *g* for 30 min (Beckman Coulter, Brea, CA, USA). The supernatants were utilized to conduct antioxidant assays, in accordance with the procedures outlined by Benjakul et al. (2014) [22] and Kantakul et al. (2024) [5].

#### 2.9.1. DPPH Radical Scavenging Activity (DPPH-RSA)

A total of 1.5 mL of the supernatant was mixed with an equivalent volume of a 0.15 mM DPPH solution in 95% methanol, bringing the total volume to 3 mL. The combined mixture was thoroughly agitated and left undisturbed for 30 min at ambient temperature in dark. Subsequently, the absorbance of the resultant mixture was read at 517 nm using a spectrophotometer. The blank sample was prepared using the same procedure; however, the DPPH solution was substituted with 95% methanol. A standard curve was constructed using Trolox within the concentration range of 10–60 μM. The DPPH-RSA was then computed after sample blank deduction using the equation derived from the standard curve and expressed as µmol of Trolox equivalents (TE)/g of dried film.

#### 2.9.2. Ferric Reducing Antioxidant Power (FRAP)

The FRAP assay was conducted by the methodology outlined by Benjakul et al. (2014) [22]. The stock solutions included 300 mM acetate buffer (pH 3.6), a 10 mM TPTZ (2,4,6-tripyridyl-s-triazine) solution in 40 mM HCl, and a 20 mM FeCl_3_·6H_2_O solution. To create the working solutions, 25 mL of acetate buffer, 2.5 mL of TPTZ solution, and 2.5 mL of FeCl_3_·6H_2_O solution were freshly mixed. The FRAP solution was created by incubating this mixture in a water bath at 37 °C for 30 min. The sample (150 µL) was mixed with the FRAP (2850 µL) solution and incubated in the dark at 25 °C for 30 min. The formation of the ferrous tripyridyltriazine complex, resulting in a colored product, was quantified by measuring the absorbance at 593 nm. For the sample blank, FeCl_3_ was excluded from the FRAP solution, and distilled water was used as a substitute. Using the Trolox solution, a standard curve was established at different amounts between 50 and 600 μM. The activity was then computed after sample blank deduction utilizing the usual curve-derived equation of the standard curve and expressed as µmol of Trolox equivalents (TE)/g of dried film.

#### 2.9.3. Oxygen Radical Absorbance Capacity (ORAC)

The ORAC assay was conducted by following the modified protocol outlined by Kantakul et al. (2024) [5]. The supernatant was diluted in 75 mM phosphate buffer (pH 7.2), ultimately achieving a concentration of 0.1 mg/mL. Then, 25 μL of this solution was dispensed into the wells of a black polystyrene microplate with 96 wells having flat bottoms. Subsequently, 50 μL of a 0.04 μM fluorescein solution, previously prepared in 75 mM phosphate buffer at pH 7.2, was introduced to each sample. The loaded microplate was incubated for 20 min at 37 °C in a Thermo Scientific Varioskan Flash Multimode Reader (Thermo Scientific Varioskan® Flash Multimode Reader, Fisher Scientific UK Ltd., Leicestershire, UK). Subsequently, the reaction was initiated by introducing 100 μL of a freshly prepared 221 mM solution of AAPH into the well. This reaction was conducted at 37 °C, and the fluorescence intensity was recorded at 5 min intervals for a total time of 90 min, using excitation and emission filters set at 485 nm and 520 nm, respectively. Similarly, a control sample was made, but instead of the sample, 75 mM phosphate buffer at pH 7.2 was utilized. The following formula was used to normalize the curves and obtain the area under the fluorescence decay curve (AUC) of the samples:

(9) $$\mathrm{AUC}=0.5+\frac{F_{2}}{F_{1}}+\frac{F_{3}}{F_{1}}+\ldots \ldots +0.5\;(\frac{F_{n}}{F_{1}})$$

where F_n_ is the final measurement and the fluorescence value at the beginning of the reaction is denoted by F_1_. The net AUC was computed by deducting the AUC of a sample or standard from the AUC of the blank. Trolox (0–100 μM) served as the reference point. The unit of measurement for the ORAC was µmol of Trolox equivalents (TE)/g of dried film.

#### 2.9.4. Metal Chelating Activity (MCA)

To conduct the MCA assay, 940 μL of supernatant of the film sample was blended with 20 μL of 2 mM FeCl_2_ and 40 μL of 5 mM ferrozine, as described by Benjakul et al. (2014) [22]. The resulting reaction mixture was left undisturbed at ambient temperature for 20 min, after which the absorbance was determined at 562 nm. EDTA at concentrations spanning from 10 to 60 μM was utilized to create a standard curve. MCA was computed and reported in terms of µmol of EDTA equivalents (EE)/g of dried film.

### 2.10. Statistical Analysis

A completely randomized design (CRD) was chosen for this study. Three triplicates of each experiment and analysis were carried out (n = 3). The Duncan multiple range test was implemented to assess the differences between means at *p* < 0.05 after ANOVA was conducted. The analysis was performed with SPSS software (Edition 28, SPSS Inc., Chicago, IL, USA).

## 3. Results and Discussion

### 3.1. Properties of Film-Forming Emulsion

#### 3.1.1. Oil Droplet Size

The *d*_32_ and *d*_43_ values of oil droplets in FFE with basil essential oil (BE) added at varying levels (50–100%, *w/w*) and different soy lecithin (SL) levels (25% and 50%, *w/w*) are shown in Table 1. *d*_32_ is associated with the specific surface area, and smaller *d*_32_ values indicates a higher specific surface area. *d*_43_ serves as an index of coalescence and flocculation, reflecting the formation of larger droplet flocs [19]. While smaller particles predominantly influence *d*_32_, larger ones more significantly affect *d*_43_ [19]. At h 0 of storage, lower *d*_32_ and *d*_43_ values were observed in FFE samples containing a lower level of BE (50%) compared to those FFE samples with a higher level of BE (>75%) (*p* < 0.05), regardless of the SL level used. On the other hand, the *d*_32_ and *d*_43_ values upsurged when the BE level in the FFE increased (*p* < 0.05). An upsurging oil percentage in emulsions led to a larger mean droplet diameter [19]. In addition, the incorporation of 100% BE and 50% SL resulted in the highest particle size (*d*_32_ and *d*_43_) (*p* < 0.05). During emulsification, SL quickly adsorbs onto the generated oil droplets, reducing surface tension, creating robust outer layers, and preventing the process of coalescence or flocculation through electrostatic or steric repulsion [23]. A high phospholipid concentration in SL has been shown to increase emulsion stability and ensure uniform oil dispersion in the emulsified film networks [17]. After 24 h of storage, increases in the *d*_32_ and *d*_43_ values were observed compared to those FFE samples with the same levels of BE and SL counterpart at 0 h of storage (*p* < 0.05), except for the FFE sample containing 100% BE and 50% SL. The collapse of the emulsion with prolonged storage might have occurred through the Oswald ripening mechanisms, flocculation-induced assembly of individual droplets, or a coalescence mechanism [24]. At extended storage periods, oil droplets tended to align closely, resulting in creaming and flocculation, which could promote emulsion coalescence. However, smaller oil droplets in the FFE exhibited greater resistance to coalescence during storage. An appropriate surfactant level (25%) enhanced the oil droplet size reduction and stabilization in emulsions, but at a high level (50%), soy lecithin might undergo self-assembly into micelles after emulsification. Consequently, the quantity of active surfactant that migrates to the interface between water and oil could be reduced [25]. Tongnuanchan et al. (2015) [26] showed that the *d*_32_ and *d*_43_ of FFE were similar across all palm oil levels immediately after preparation but slightly increased with longer storage times, particularly after 12 h.

#### 3.1.2. Flocculation Factor (F_f_) and Coalescence Index (C_i_)

The flocculation factor (*F*_f_) and coalescence index (*C*_i_) of all FFE samples during 0 h and 24 h of storage are shown in Table 1. A higher *F*_f_ was observed as higher levels of BE and SL were used (*p* < 0.05), irrespective of the storage period. Typically, this was consistent with the elevated *d*_32_ and *d*_43_ values observed in these samples. However, a high *F*_f_ value was observed in FFE sample emulsified with 100% BE and 50% SL, irrespective of the storage period. The results aligned with the increases in *d*_32_ and *d*_43_ observed in these samples after storage. It was indicated that the oil droplets in the FFE formed clusters through flocculation. Droplet flocculation in emulsions typically occurs due to the attraction between oil droplets, but it can be reduced when steric repulsion surpasses these attractive forces [27].

Following 24 h of storage, the *C*_i_ was measured for all FFE samples (*p* < 0.05). Coalescence indicates emulsion instability. The larger *d*_43_ value shows the collapse of oil droplets responsible for the increase in *C*_i_ [28]. The highest *C*_i_ was observed in the FFE emulsified with BE (100%) and SL (50%). The increased *C*_i_ of the droplets in FFE containing high levels of BE (100%) was likely due to the closer gathering of scattered oil droplets in the presence of more oil. Moreover, soy lecithin formed a protective film on the droplets’ surfaces. As a consequence, the interfacial tension was reduced, while coalescence was prevented [25]. Thus, the basil essential oil and soy lecithin levels significantly affected the emulsion stability.

#### 3.1.3. Confocal Laser Scanning Microscopy (CLSM) Images

**Figure 1:**
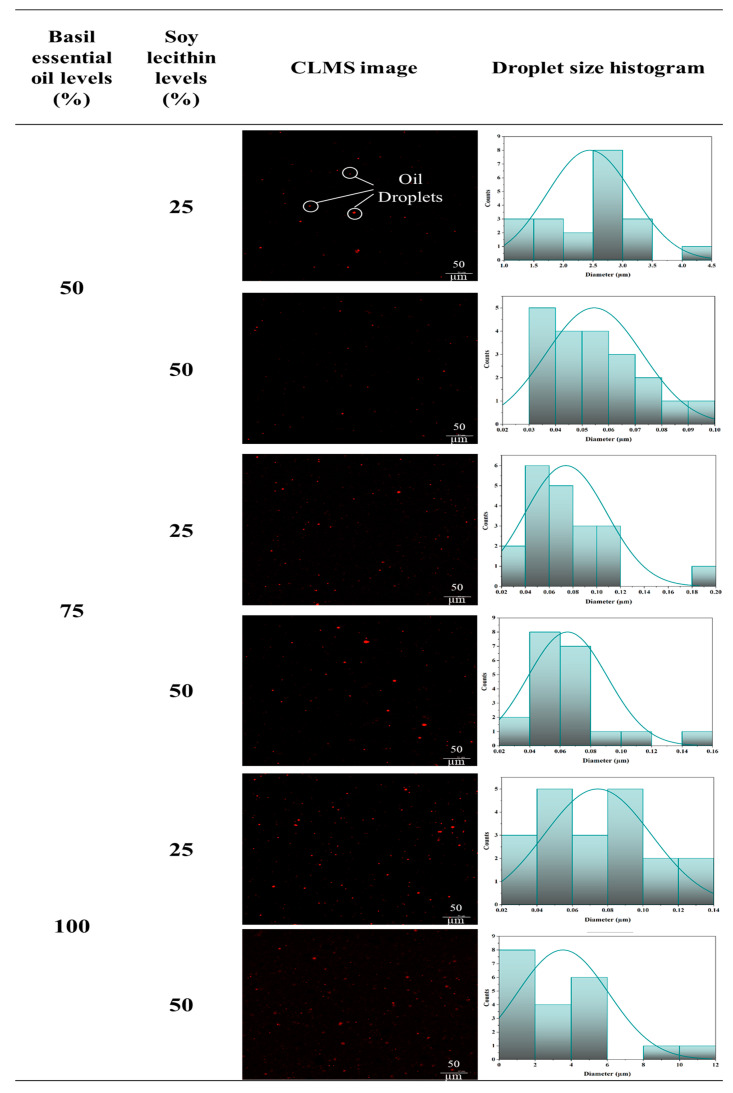
CLSM images and histograms of the particle sizes of film-forming emulsions based on co-precipitated proteins from Bambara groundnut protein isolate and fish skin acid-soluble collagen emulsified with different levels of basil essential oil and soy lecithin. Magnification 200×.

Figure 1 presents CLSM images of FFE samples incorporating BE at levels of 50%, 75%, and 100% (*w/w*, according to protein), as well as SL at levels of 25% and 50% (*w/w*, according to BE). CLSM micrographs showed the red-colored oil droplets, contrasting with the dark background of the unstained protein continuous phase. Notably, BE was distributed in the FFE, and the number of small droplets increased with higher levels of BE. The histogram (Figure 1) also presented smaller droplet sizes distributed uniformly. Moreover, the aggregation of oil droplets into larger droplets was observed at 100% BE incorporation, irrespective of SL levels. The result aligned well with the findings of Tongnuanchan et al. (2015) [26]. This result suggested that a lower level of BE (50–75%) could form a stable emulsion. Tongnuanchan et al. (2015) [26] stated that reducing the oil content in the sample led to smaller droplet sizes. The primary role of the surfactant is to prevent droplet re-coalescence by quickly adsorbing at oil droplets and stabilizing the interface. In this context, excess surfactant in the continuous phase interrupted their adsorption at the oil/water interface [25]. Thus, the drops of oil could spread out uniformly in the FFE with sufficient amounts of basil essential oil and soy lecithin.

### 3.2. Properties and Characteristics of the Films

#### 3.2.1. Appearance and Thickness

**Figure 2:**
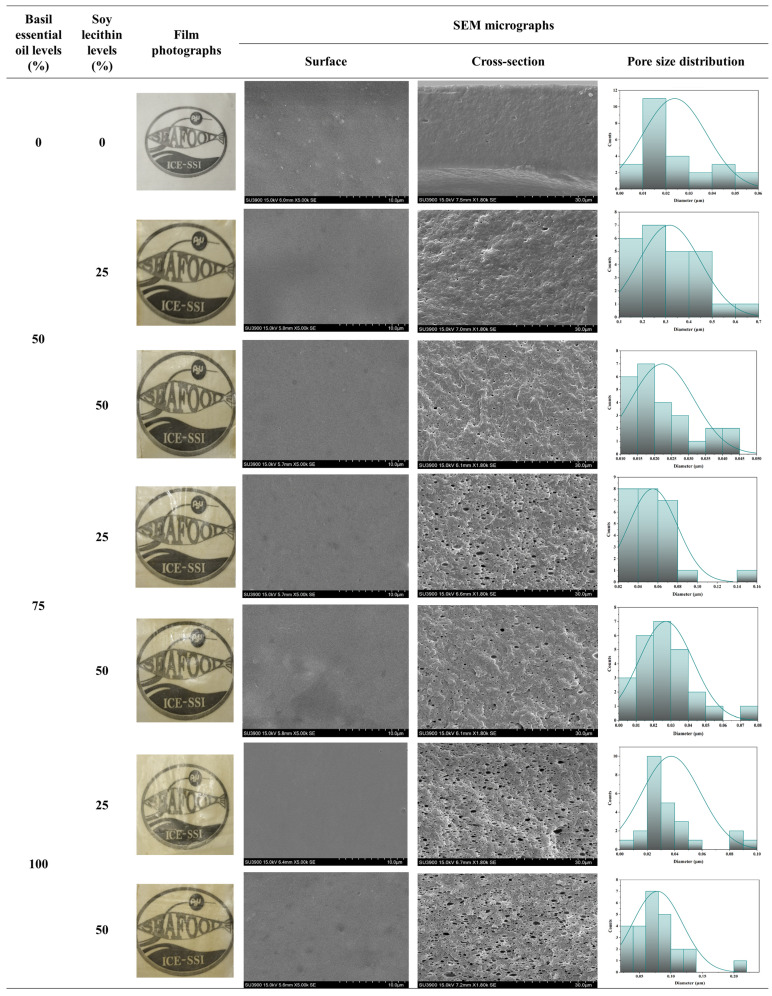
Film photographs, scanning electron microscopy (SEM) micrographs of the surface (magnification: 5000×) and cross-section (magnification: 1800×) and pore size distribution histograms (cross-section) of emulsified films based on co-precipitated proteins from Bambara groundnut protein isolate and fish skin acid-soluble collagen emulsified with different levels of basil essential oil (BE) and soy lecithin (SL). Control, film without BE and SL; BE_50%_-SL_25%_, BE_50%_-SL_50%_, BE_75%_-SL_25%_, BE_75%_-SL_50%_, BE_100%_-SL_25%_, and BE_100%_-SL_50%_, emulsified films incorporating BE at levels of 50–100% and SL at levels of 25% and 50%.

Figure 2 shows photographs of co-precipitated protein (CPP) films incorporated with and without different levels of basil essential oil (BE) and soy lecithin (SL). The control CPP film without BE and SL was transparent and clear. A yellowish color with a uniform appearance and no oil exudate on the film surface were observed for all CPP films incorporated with BE and SL. The intensity of the yellow color was increased as the levels of BE and SL increased. The darkest yellow color was obtained for the emulsified film containing 100% BE and 50% SL. As asserted by Ribeiro-Santos et al. (2017) [29], higher levels of essential oils (cinnamon, basil, and rosemary) resulted in a more yellowish color and increased the opaqueness of a whey protein concentrate-based film. The additions of surfactants (soy lecithin, Tween-20, and Tween-80) and essential oils (basil and citronella) changed the color and transparency of the resulting gelatin films [17].

The thickness of co-precipitated protein (CPP) films incorporated with and without different levels of BE (50%, 75%, and 100% *w/w*, based on the CPP content) and SL (25% and 50% *w/w*, based on the BE content) is demonstrated in Table 2. Overall, all of the films had a thickness between 0.052 and 0.118 mm. The films with higher BE and SL levels were thicker than the control CPP film (absence of BE and SL) (*p* < 0.05). The interaction between protein chains can be hindered by smaller BE droplets that penetrate the film network. The compact network was lost and the subsequent decrease in the ordered arrangement of the protein chains led to the protruding structure, as shown by the thicker film [8]. In addition, the thickness of films comprising 50% SL was higher than that of films containing 25% SL (*p* < 0.05) when identical levels of basil essential oil were used. Nilsuwan et al. (2016) [25] documented the differences in the fish skin gelatin film thickness caused by the different SL contents in the formulations. Therefore, the different levels of BE and SL had impacts on the thickness of the emulsified CPP film.

#### 3.2.2. Mechanical Properties

Table 2 presents the tensile strength (TS) and elongation at break (EAB) of CPP films emulsified with and without varying levels of BE (50%, 75%, and 100% *w/w*, based on the CPP content) and SL (25% and 50% *w/w*, based on the BE content). The additions of BE and SL significantly altered the mechanical properties of the films. Films containing BE exhibited a lower TS but higher EAB than those without BE and SL (*p* < 0.05). The TS of the emulsified CPP films decreased from 5.78 to 0.95 MPa, while the EAB increased from 93.96 to 221.9%. These alterations became more pronounced with increasing BE and SL levels. This trend aligned with Zhao et al. (2023) [30], who observed a decreased TS and increased EAB in soy protein isolate emulsion (SPIE) films combined with a range of pre-emulsions made from distinct emulsifiers. The decrease in TS and increase in EAB can be attributed to basil essential oil, which contains various compounds that intercalate between protein chains, thereby reducing the intramolecular and intermolecular bonding of the film matrix [30]. The lowest TS and highest EAB were found in the film containing 100% BE and 50% SL (*p* < 0.05). SL has a lower HLB value (4.0), which indicates a greater hydrophobicity that allows its polar groups to bind with proteins and its non-polar groups to interact with essential oils, thereby modifying the film’s properties. Silva et al. (2021) [31] additionally stated that oils, such as palm oil and essential oils from clove and oregano, acted as plasticizers to enhance the extensibility of protein films. Thus, the incorporation of BE and SL at varying levels in CPP films directly influenced the mechanical properties of the resulting films.

#### 3.2.3. Water Vapor Permeability (WVP)

WVP measures water vapor that passes through biopolymer films. A low WVP is desirable as it prevents water movement from the surroundings to packed foods [5]. The WVP of the CPP film incorporating basil essential oil (BE) and soy lecithin (SL) at varying levels is presented in Table 2. Notably, CPP films containing varying levels of BE and SL exhibited significantly lower WVPs than the film without BE and SL (*p* < 0.05). Specifically, the WVP was decreased markedly from 5.12 in the control film to 4.76, 3.56, and 2.54 × 10^−11^ g m/m^2^ s Pa (*p* < 0.05) in the emulsified CPP films including BE at levels of 50%, 75%, and 100% in the presence of 25% SL, and to 4.74, 3.50, and 2.51 × 10^−11^ g m/m^2^ s Pa in the presence of 50% SL, respectively. This result showed that the addition of nonpolar or hydrophobic substances, such as BE, likely enhanced the hydrophobicity of the films, thus reducing water vapor adsorption and penetration. Similarly, a gelatin film from fish skin with the addition of BE (100% *w/w*, based on the protein content), SL (25% *w/w*, based BE), and glycerol (30%) had the lowest WVP values of 0.71× 10^−11^ g m/m^2^ s Pa [8] and 1.20 × 10^−11^ g m/m^2^ s Pa [10]. A noticeable reduction in the WVP of chitosan–basil oil films was observed at 0.5% basil oil [32]. The size of the oil droplets in the film-forming solution had a negative correlation with the water vapor barrier properties. However, water vapors’ route tortuosity was increased by evenly spaced tiny droplets, which lowered the WVP. Furthermore, at the same BE level, the WVP was not significantly changed as the SL level was increased (*p* > 0.05). Moreover, Tongnuanchan et al. (2013) [10] found that different types of surfactants affected the WVP of fish gelatin films, in which SL yielded the lowest WVP. This was likely because soy lecithin facilitated a uniform oil droplet distribution within the film matrix, reducing the diffusion of water molecules. Additionally, the inclusion of an emulsifier might have reduced the humidity content of the films by facilitating hydrogen bond interactions between the hydrocolloid and the polar groups of the emulsifier, thereby decreasing the availability of polar groups to interact with water molecules [33]. Additionally, the hydrophobic domains of phospholipids or lecithin may contribute to reducing water vapor transfer, as evidenced by the lower WVP. Therefore, the addition of BE, particularly in combination with SL, effectively strengthened the characteristics of the WVP of emulsified CPP films.

#### 3.2.4. Color

Table 2 shows the color attributes (L*, a*, b*, and ΔE*) of emulsified CPP films containing various levels of BE and SL. Overall, the amounts of BE and SL significantly affected the film color. As the levels of BE and SL increased, lower L* (lightness) and a* values were observed in emulsified CPP films, accompanied by correspondingly higher b* and ΔE* values, compared to the control film (*p* < 0.05). This finding was consistent with the observations of Arfat et al. (2014) [34], who reported that fish protein isolate/fish skin gelatin films in the presence or absence of 3% ZnO nanoparticles showed higher b* and ΔE* values, along with lower L* and a* values with increasing levels of basil essential oil. The color changes (yellowness) in the resulting films were probably due to the coloring components present in the essential oil and smaller oil droplets dispersed uniformly in the film matrix. Among the various levels of BE and SL, the emulsified film containing 100% BE and 50% SL showed the lowest L* (78.66) and a* (−1.96) along with the highest b* (35.30) and ΔE* (37.55) values (*p* < 0.05). Conversely, 50% BE and 25% SL provided the films with the highest L* (81.63) and a* (−1.42), as well as the lowest b* (24.74) and ΔE* (26.68) (*p* < 0.05). Rashid et al. (2023) [35] found that the addition of essential oils (curcumin and orange) increased the pullulan-based film’s yellowness (b* value from 6.55 to 30.88) but decreased lightness (L* value from 86.39 to 83.39). Tongnuanchan et al. (2014) [8] also reported that fish skin gelatin films incorporated with Plai essential oil exhibited the highest b* (20.25) and ΔE* (20.39) values, along with the lowest L* (88.42) value, compared to those added with basil and lemon essential oils. In addition, increasing the soy lecithin levels from 25% to 50% in emulsified CPP films resulted in lower L* values with simultaneously higher b* and ΔE* values, indicating increased yellowness of the emulsified films. The resulting films with higher b* and lower L* values were mostly brought by the brownish-yellow color of soy lecithin. This finding was consistent with Tongnuanchan et al. (2013) [10], who reported that fish skin gelatin films incorporated with leaf essential oil tend to be yellowish when soy lecithin is used as a surfactant. Thus, the levels of BE and SL prominently influenced the color of the emulsified CPP film.

#### 3.2.5. Light Transmission

**Figure 3:**
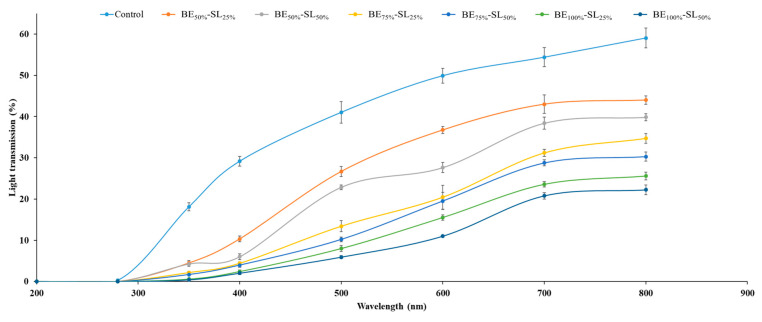
Light transmission curves of emulsified films based on co-precipitated proteins from Bambara groundnut protein isolate and fish skin acid-soluble collagen emulsified with different levels of basil essential oil (BE) and soy lecithin (SL). Control, film without BE and SL; BE_50%_-SL_25%_, BE_50%_-SL_50%_, BE_75%_-SL_25%_, BE_75%_-SL_50%_, BE_100%_-SL_25%_, and BE_100%_-SL_50%_, emulsified films incorporating BE at levels of 50–100% and SL at levels of 25% and 50%.

The light transmission values at wavelengths from 200 to 800 nm in the UV and visible range of emulsified CPP films with different BE and SL levels are shown in Figure 3. All films, including the control film and the emulsified CPP films, exhibited excellent UV light barrier properties at 200 and 280 nm, consistent with our previous study [7]. Protein-based films exhibited a superior UV light barrier capacity, attributed to their abundant aromatic amino acids that efficiently absorb UV light [19]. Emulsified CPP films incorporated with BE and SL exhibited lower visible light transmission in the range of 350–800 nm compared to the control film, and transmission decreased with increasing BE and SL levels. Films with 100% BE and 50% SL exhibited the highest barrier properties against light transmission at wavelengths of 350–800 nm. The light scattering of the lipid droplets dispersed in the protein network could have contributed to the reduced light transmission [19]. This result agreed with the findings of the fish gelatin films incorporated with essential oils and surfactants, which showed an excellent UV barrier and a good barrier to visible light in the 350–800 nm range [10]. When the proportion of soy lecithin was increased from 25% to 50%, the barrier property was more pronounced, irrespective of the various levels of basil essential oil used. The emulsified CPP films incorporated with BE and SL therefore could reinforce the UV light barrier properties of the resulting emulsified film.

#### 3.2.6. Scanning Electron Microscopy (SEM)

The SEM micrographs of the surface (5000×) and cross-sections (1800×) (Figure 2) of the developed CPP films without and with varying levels of BE and SL are shown. The control film had a smooth surface with some particles. It might be related to partial phase separation. Moreover, a uniform and smooth surface was observed for all emulsified films, regardless of the levels of BE and SL used. This indicated the effective compatibility and small droplets of the added active ingredients (BE) dispersed into the protein matrix. Cazón et al. (2021) [36] reported that SEM images showed a smooth surface without cracks due to the uniform distribution of tea tree essential oil in the chitosan film matrix in the presence of soy lecithin. The structural arrangement of the components influences the final microstructure of the packaging material, which can be altered by coalescence, creaming, and droplet flocculation during the drying process [37]. In addition, whey protein isolate-based nanoemulsion films with orange peel essential oil showed a smoother surface with less roughness and irregularities compared to emulsion and control films [38].

Furthermore, the increase in the number of pores in the cross-sections of films augmented with basil essential oils and soy lecithin levels was noticeable, indicating phase separation and a less compacted structure. The pore size distribution was calculated using the SEM cross-section images and ImageJ software (Version 1.54g) and is presented in Figure 2. The different sizes and number of cavities were more likely due to BE droplets dispersed in the protein matrix, with the morphological changes in the films related to the miscibility of the components and the incorporation of high BE concentrations. These results suggested that increasing the soy lecithin concentration possibly facilitated the retention of BE droplets in the film matrix during the process of drying, and the resulting film’s structure became coarse. This finding was consistent with Ali et al. (2019) [39], who documented that the addition of increased levels of glycerol and squalene to fish skin gelatin films resulted in a coarser film network. Moreover, these sponge-like structures formed due to the uneven dispersion of hydrophobic BE from the aqueous phase during casting and drying processes. On the other hand, the control film (without BE and SL) was more compact than the other films. The cross-sectional image showed voids with different sizes due to varying droplets of oregano essential oil (OEO) dispersed by Tween 80 in the soy protein concentrate matrix. Morphological changes in the films were related to the immiscibility of the components and the incorporation of high OEO concentrations [40]. Therefore, the levels of BE and SL had vital impacts on the surface and cross-section of the resulting films.

#### 3.2.7. Sealability

Materials used for biodegradable packaging should be sealable when heated. The surfaces of the two films were melted due to the heat generated during heat sealing. This might enhance the interactions between the film surfaces that come into contact with each other, thus providing a strong seal to the film [20]. The seal must be strong enough to safeguard and maintain the products in the package during handling or storage [20]. This study investigated the seal strength (SS) and seal efficiency (SE) of emulsified CPP films without and with various levels of BE and SL. All films were heat sealable (Table 3) but showed different SSs and SEs (*p* < 0.05). The SS and SE of the emulsified CPP film showed a distinct pattern with the different levels of BE and SL. Notably, the control film (absence of BE and SL) showed the highest SS (222.44 N/m) with lowest SE (79.82%) (*p* < 0.05). The emulsified CPP film with 100% BE and 50% SL exhibited the lowest SS (107.56 N/m) but the highest SE (145.88%) among all emulsified films. As BE and SL were incorporated, a continuous increase in SS was observed, particularly when higher levels of BE and SL were used. Tongnuanchan et al. (2016) [20] documented that a fish skin gelatin emulsified film had a lower SS but higher SE than the control film (without oil). Various elements in basil essential oil might have distinct effects on altering the film’s protein network. Basil essential oil, which is rich in monoterpene hydrocarbons as its major components [10], could disrupt or interact with protein molecules through hydrophobic interactions. Aldehydes, ketones, and phenols are among several chemicals found in essential oils, which are concentrated hydrophobic liquids [11]. The hydroxyl and hydrophobic groups of polyphenols in basil essential oil interact with protein chains via hydrogen bonding and hydrophobic interactions, forming a rigid protein network. This rigidity reduces melting during heat sealing and impedes molecular interdiffusion across the molten film interface, leading to lower seal strength and efficiency. Rosenbloom and Zhao (2021) [41] reported that the addition of oleic acid (0 or 0.25%) and DL-α-tocopherol acetate (0.1 or 0.2%) to soy protein isolate-based films significantly increased the seal strength up to 143 N/m. Furthermore, CPP films emulsified with BGPI and ASC and different levels of BE and SL showed more than one mode of failure. The mode of failure reflects the quality of heat sealing [20]. Adhesive failure (mode I) was observed in all films, suggesting that the seal was formed due to incomplete protein fusion, resulting in a weaker seal. This mode of failure implies inadequate sealing and the weak seals were caused by chain entanglement defects in a limited number of film samples during the test. A similar result was also reported for gelatin films incorporating BE and SL [20]. This outcome was linked to the film’s lowest seal strength and efficiency. Moreover, the cohesive seal failure (mode II) was observed in emulsified films containing 100% BE and SL at both 25% and 50%. In general, sealed films exhibiting this mode of failure tended to have greater elongation and strength values. Greater molecular diffusion and entanglement in the area of the seal made it difficult to separate the film and finally resulted in elongation [42]. Therefore, the various levels of BE and SL played a crucial role in the sealing properties of emulsified CPP films.

#### 3.2.8. FTIR

**Figure 4:**
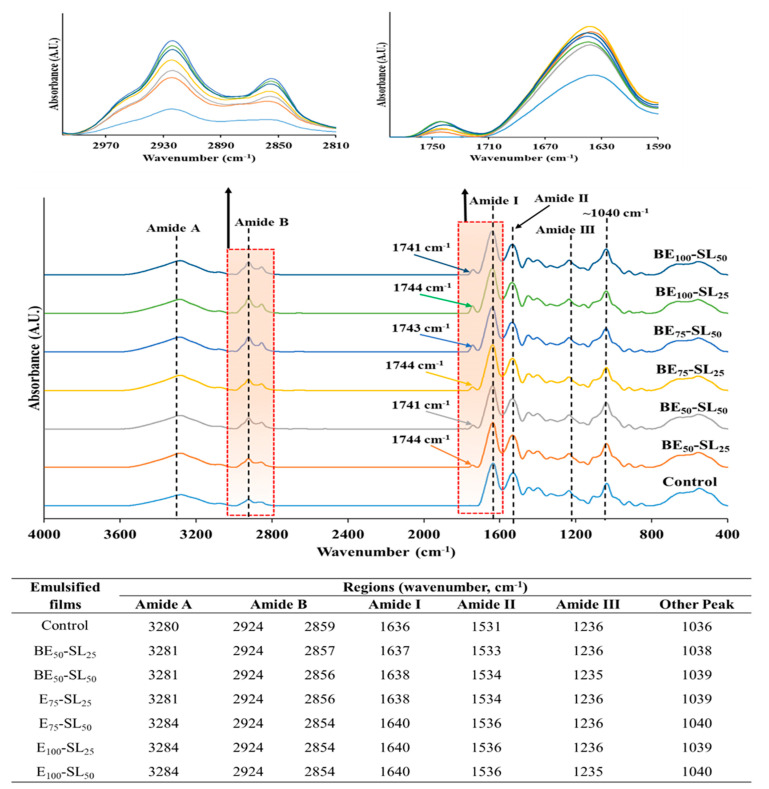
FTIR spectra of emulsified films based on co-precipitated proteins from Bambara groundnut protein isolate and fish skin acid-soluble collagen emulsified with different levels of basil essential oil (BE) and soy lecithin (SL). Control, film without BE and SL; BE_50%_-SL_25%_, BE_50%_-SL_50%_, BE_75%_-SL_25%_, BE_75%_-SL_50%_, BE_100%_-SL_25%_, and BE_100%_-SL_50%_, emulsified films incorporating BE at levels of 50–100% and SL at levels of 25% and 50%.

Figure 4 presents the FTIR spectra of control and emulsified CPP films. All CPP films showed similar major peaks (amide A, B, I, II, and III). The peak amplitudes varied with varying levels of BE and SL. The peak at wavenumbers of 3280–3284 cm^−1^ (amide A) appeared for all CPP film samples, reflecting intramolecular and intermolecular hydrogen bonding along with N-H and O-H stretching vibrations [5]. Notably, these peaks shifted from 3280 cm^−1^ in the control film (without BE and SL) to 3284 cm^−1^ in the CPP films emulsified with 100% BE, regardless of the level of SL used. This result indicated a reduction in protein–protein interactions due to the presence of BE and SL compared to the control film. Typically, a shift of amide A to a lower wavenumber indicated augmented hydrogen bonding between protein molecules [25]. The decreased protein–protein interactions could be attributed to the more uniform distribution of BE droplets within the film matrix, which were stabilized by SL [17]. Moreover, the amide B band for all film samples was observed at 2924 cm^−1^ and 2859–2854 cm^−1^, which represented the symmetrical and asymmetrical stretching vibrations of aliphatic C-H bonds in CH_2_ and CH_3_ groups, respectively [25]. Such stretching vibration bands are commonly associated with most lipids and other hydrophobic substances [25]. The shift to lower wavenumbers (2857–2854 cm^−1^) observed in emulsified films compared to control film (2859 cm^−1^) indicated the presence of hydrophobic substances. Both peaks had an increase in amplitude with increasing levels of BE and SL.

Furthermore, essential oils contain primary chemical groups such as aldehyde, ketone, ester, ethers, and phenols [11]. The absorption of a carbonyl (C=O) group representing these compounds was observed in all emulsified CPP films at wavenumbers ranging from 1741 to 1744 cm^−1^, whereas no such peak was detected in the control film. Similarly, carbonyl group peaks (C=O) were reported in soy protein films with soybean oil and olive oil [43] and fish gelatin incorporated with basil and citronella essential oils in combination with different surfactants [17]. The higher peak amplitude of amide B was also obtained for all emulsified films compared to the control film. The amide I band of the control film and all emulsified CPP films was attained at wavenumbers ranging from 1636 to 1640 cm^−1^, corresponding to C=O stretching vibrations associated with CN stretching and NH bending or COO- group coupled with hydrogen bonding [5]. The amide I band of the emulsified CPP films was shifted to higher wavenumber (1640 cm^−1^) when BE and SL were incorporated compared to the control film (1636 cm^−1^). This shift could be due to a reduction in the interactions between the protein chains that was to some extent caused by the addition of BE and SL [39]. The amide II region (N-H bending, combined with C-N) was present in the CPP control film at a wavenumber of 1531 cm^−1^. However, with increasing levels of BE and SL in emulsified CPP films, the wavenumber of this peak gradually increased to a higher wavenumber. This shift suggested the interruption of protein chains in the film matrix by BE and SL. Similarly, the wavenumber of the amide II band increased slightly in films incorporating a squalene-rich fraction from shark liver (10 and 25%) and palm oil (25%), shifting from 1547 cm^−1^ in the control film to 1548, 1548, and 1550 cm^−1^, respectively [39]. The shift in the amide I and II bands to lower wavenumbers in the emulsified CPP films, particularly at higher levels of BE and SL, indicated the weakness of protein interactions in the film matrix. This result was aligned with the lower TS of the resulting film, as shown in Table 2. The amide III bands, reflecting in-plane vibrations of C-N groups in bound amides and absorptions caused by CH_2_ wagging vibrations from the glycine backbone and proline side chains [26], were found similarly in all films in the wavenumber range of 1235–1236 cm^−1^. Additionally, a band at wavenumbers between 1036 and 1040 cm^−1^ was observed in all CPP films and corresponded to the -OH group coupled to –CH_2_ of glycerol [7], which was used as a plasticizer. Therefore, the incorporation of BE and SL directly affected the molecular interactions of the protein chains within the CPP film matrix.

### 3.3. Antioxidant Activities

The antioxidant activities, including DPPH-RSA, FRAP, ORAC, and MCA, of the control and emulsified CPP films with different levels of BE and SL are presented in Table 3. All antioxidant activities of the emulsified CPP films upsurged with increasing BE and SL levels (*p* < 0.05). The lowest values of those activities were found for the control film (absence of BE and SL) (*p* < 0.05), regardless of the assays used. Generally, numerous essential oils are known to possess significant antioxidant properties [11]. Moreover, the presence of bioactive compounds (linalool, estragole, methyl cinnamate, eucalyptol, and eugenol) in basil essential oils exhibited good antioxidant power [12]. Additionally, soy lecithin also exhibited excellent antioxidant properties in the resulting films [44]. Among all emulsified films, the film with 100% BE and 50% SL showed the highest DPPH-RSA (60.45 µmol TE/g dried film) and FRAP (33.10 µmol TE/g dried film) (*p* < 0.05). No significant differences between the ORAC and MCA of emulsified films with 100% BE at both SL levels (25% and 50%) were observed (*p* > 0.05). The control film had DPPH-RSA (2.69 µmol TE/g dried film), FRAP (5.86 µmol TE/g dried film), ORAC (377.74 µmol TE/g dried film) and MCA (528.15 µmol EE/g dried film). These results could be due to the fact that the Bambara groundnut protein isolate film had antioxidant activity [5] and a pure collagen film also exhibited some antioxidant activity [45]. Emulsified CPP films could therefore have the potential to be used as antioxidant films for food applications. Additionally, Tongnuanchan et al. (2013) [10] documented that a film containing BE showed the highest DPPH and ABTS radical scavenging activities compared to the films incorporating other essential oils (lemongrass, citronella, and kaffir lime). The activity was further enhanced in films by the use of soy lecithin alongside essential oils.

## 4. Conclusions

The incorporation of BE and SL at varying levels influenced the properties of FFE and emulsified CPP films. The oil particle sizes (*d*_32_ and *d*_43_) were increased with augmented levels of BE and SL, irrespective of the storage period. Emulsified CPP films showed an increased thickness and EAB when the BE and SL contents increased, while the TS and WVP decreased. The incorporation of BE and SL directly affected the film color (dark yellow) and increased the light barrier properties. All CPP films were sealable, and the sealing properties improved after the addition of BE and SL. The FTIR spectra elucidated intermolecular interactions between the functional groups of the BE and the amino and hydroxyl groups in the emulsified CPP film network, but hampered the interaction between protein chains. Emulsified CPP films incorporated with appropriate levels of BE and SL showed good antioxidant activities. Therefore, the addition of BE (100%) and SL (25%) in the CPP film showed satisfactory properties and antioxidant activities, and it could be used as active packaging.

## Figures and Tables

**Table 1:** Oil droplet size and emulsion stability of film-forming emulsions based co-precipitated proteins from Bambara groundnut protein isolate and fish skin acid-soluble collagen emulsified with different levels of basil essential oil and soy lecithin.

Basil Essential Oil Levels (%)	Soy Lecithin Levels (%)	Storage Time (h)	*d*_32_ (µm)	*d*_43_ (µm)	*F*_f_	*C*_i_
50	25	0	0.314 ± 0.01 *^f^	0.568 ± 0.01 ^c^	4.348 ± 0.01 ^d^	-
		24	0.402 ± 0.01 ^E^	0.623 ± 0.01 ^F^	10.844 ± 0.31 ^C^	8.754 ± 0.54 ^e^
	50	0	0.392 ± 0.03 ^e^	0.598 ± 0.01 ^c^	4.441 ± 0.05 ^d^	-
		24	0.609 ± 0.01 ^D^	0.692 ± 0.01 ^E^	10.998 ± 0.03 ^C^	13.596 ± 0.53 ^d^
75	25	0	0.514 ± 0.01 ^d^	0.780 ± 0.01 ^b^	4.770 ± 0.06 ^c^	-
		24	0.674 ± 0.01 ^C^	0.925 ± 0.01 ^D^	11.368 ± 0.06 ^B^	15.684 ± 0.04 ^c^
	50	0	0.575 ± 0.02 ^c^	0.792 ± 0.02 ^b^	4.859 ± 0.02 ^c^	-
		24	0.698 ± 0.01 ^B^	0.951 ± 0.01 ^C^	11.445 ± 0.12 ^B^	16.194 ± 0.22 ^c^
100	25	0	0.696 ± 0.02 ^b^	0.925 ± 0.02 ^a^	5.298 ± 0.09 ^b^	-
		24	0.747 ± 0.01 ^A^	1.141 ± 0.01 ^B^	12.026 ± 0.02 ^A^	18.941 ± 0.73 ^b^
	50	0	0.888 ± 0.04 ^a^	0.898 ± 0.06 ^a^	5.520 ± 0.06 ^a^	-
		24	0.750 ± 0.02 ^A^	1.198 ± 0.01 ^A^	12.287 ± 0.01 ^A^	20.908 ± 0.93 ^a^

* Values are means ± SDs (*n* = 3). Different lowercase superscript letters in the same column at the same storage time (0 h) indicate significant differences (*p* < 0.05). Different uppercase superscript letters in the same column at the same storage time (24 h) indicate significant differences (*p* < 0.05). *F*_f_—flocculation factor; *C*_i_—coalescence index.

**Table 2:** Thickness, mechanical properties, water vapor permeability, and color of emulsified films based on co-precipitated proteins from Bambara groundnut protein isolate and fish skin acid-soluble collagen emulsified with different levels of basil essential oil and soy lecithin.

Basil Essential Oil Levels (%)	Soy Lecithin Levels (%)	Thickness (mm)	TS (MPa)	EAB (%)	WVP (×10^−11^ g m/m^2^ s Pa)	L*	a*	b*	ΔE*
Without BE	Without SL	0.052 ± 0.001 *^f^	5.78 ± 0.45 ^a^	93.96 ± 9.46 ^e^	5.12 ± 0.24 ^a^	84.90 ± 0.20 ^a^	−0.77 ± 0.07 ^a^	11.33 ± 0.63 ^g^	13.40 ± 0.50 ^g^
50	25	0.085 ± 0.004 ^e^	4.44 ± 0.46 ^b^	105.41 ± 11.36 ^de^	4.76 ± 0.12 ^b^	81.63 ± 0.77 ^b^	−1.42 ± 0.08 ^b^	24.74 ± 0.24 ^f^	26.68 ± 0.17 ^f^
	50	0.093 ± 0.008 ^d^	3.60 ± 0.58 ^c^	115.63 ± 12.97 ^d^	4.74 ± 0.31 ^b^	81.26 ± 0.34 ^b^	−1.54 ± 0.05 ^c^	27.47 ± 0.34 ^e^	29.32 ± 0.34 ^e^
75	25	0.104 ± 0.004 ^c^	2.72 ± 0.3 ^d^	151.16 ± 19.08 ^c^	3.56 ± 0.16 ^c^	80.14 ± 0.21 ^c^	−1.72 ± 0.08 ^d^	29.92 ± 0.40 ^d^	32.0 ± 0.34 ^d^
	50	0.106 ± 0.002 ^c^	2.03 ± 0.39 ^e^	178.58 ± 23.75 ^b^	3.50 ± 0.05 ^c^	79.80 ± 0.63 ^cd^	−1.83 ± 0.04 ^e^	31.24 ± 0.73 ^c^	33.36 ± 0.56 ^c^
100	25	0.113 ± 0.003 ^b^	1.23 ± 0.18 ^f^	208.52 ± 17.53 ^a^	2.54 ± 0.09 ^d^	78.92 ± 1.05 ^de^	−1.91 ± 0.03 ^ef^	34.03 ± 0.39 ^b^	36.28 ± 0.57 ^b^
	50	0.118 ± 0.002 ^a^	0.95 ± 0.12 ^f^	221.9 ± 18.12 ^a^	2.51 ± 0.1 ^d^	78.66 ± 0.95 ^e^	−1.96 ± 0.03 ^f^	35.30 ± 0.58 ^a^	37.55 ± 0.73 ^a^

* Values are means ± SDs (*n* = 3). Different lowercase superscript letters in the same column indicate significant differences (*p* < 0.05). TS—tensile strength; EAB—elongation at break; WVP—water vapor permeability, BE—basil essential oil; SL—soy lecithin.

**Table 3:** Seal strength, seal efficiency, and mode of failure, and antioxidant activities of emulsified films of co-precipitated proteins from Bambara groundnut protein isolates and fish skin acid-soluble collagen emulsified with different levels of basil essential oil and soy lecithin.

Basil Essential Oil Levels (%)	Soy Lecithin Levels (%)	Sealed Films	Seal Strength (N/m) *	Seal Efficiency (%)	Mode of Failure	DPPH-RSA **	FRAP **	MCA ***	ORAC **
Without BE	Without SL	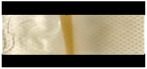	222.44± 12.61 *^a^	79.82 ± 7.59 ^f^	II, I	2.69 ± 0.03 *^f^	5.86 ± 0.74 ^g^	528.15 ± 16.97 ^e^	377.74 ± 4.45 ^d^
50	25	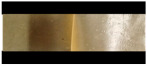	191.44 ± 10.81 ^b^	87.30 ± 5.56 ^ef^	I	18.66 ± 0.12 ^e^	13.48 ± 0.29 ^f^	568.89 ± 22.22 ^d^	855.90 ± 22.54 ^c^
	50	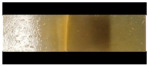	177.88 ± 11.63 ^c^	93.71 ± 5.69 ^e^	I	19.86 ± 0.23 ^e^	15.78 ± 0.53 ^e^	597.04 ± 14.29 ^c^	891.51 ± 24.16 ^bc^
75	25	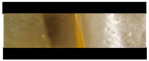	159.38 ± 9.27 ^d^	114.97 ± 8.14 ^d^	I	28.31 ± 0.46 ^d^	20.84 ± 0.39 ^d^	619.63 ± 5.70 ^bc^	933.99 ± 31.89 ^b^
	50	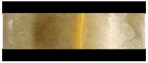	143.63 ± 7.08 ^e^	123.80 ± 10.24 ^c^	I	37.42 ± 5.17 ^c^	24.11 ± 0.35 ^c^	639.63 ± 11.98 ^b^	941.46 ± 5.81 ^b^
100	25	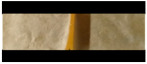	121.81 ± 9.96 ^f^	136.76 ± 8.35 ^b^	I, II	51.97 ± 2.29 ^b^	30.18 ± 0.40 ^b^	669.26 ± 3.39 ^a^	1128.30 ± 45.95 ^a^
	50	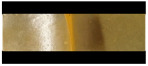	107.56 ± 5.66 ^g^	145.88 ± 10.17 ^a^	I, II	60.45 ± 2.73 ^a^	33.10 ± 0.55 ^a^	676.30 ± 6.41 ^a^	1140.22 ± 45.08 ^a^

* Values are given as the means ± SDs (*n* = 3). Different lowercase superscript letters in the same column indicate significant differences (*p* < 0.05). I = adhesive failure; II = cohesive failure; DPPH-RSA = DPPH radical scavenging activity; FRAP = ferric reducing antioxidant power; MCA = metal chelating activity; ORAC = oxygen radical absorbance capacity; ** the unit of the values is µmol of TE/g of dried film; *** the unit of the values is µmol of EE/g of dried film; TE = Trolox equivalents; EE = EDTA equivalents.

## Data Availability

The data are contained within the article.
